# Rapid detection of goose astrovirus genotypes 2 using real-time reverse transcription recombinase polymerase amplification

**DOI:** 10.1186/s12917-023-03790-2

**Published:** 2023-11-07

**Authors:** Haiqin Li, Yujun Zhu, Chunhe Wan, Zhangzhang Wang, Lei Liu, Meifang Tan, Fanfan Zhang, Yanbing Zeng, Jiangnan Huang, Chengcheng Wu, Yu Huang, Zhaofeng Kang, Xiaoqiao Guo

**Affiliations:** 1 Institute of Animal Husbandry and Veterinary Medicine, Jiangxi Academy of Agricultural Sciences, Nanchang, 330200 Jiangxi China; 2 Jiangxi Provincial Key Laboratory for Animal Health, College of Animal Science and Technology, Jiangxi AgriculturalUniversity, Nanchang, China; 3 Guangdong laboratory animals monitoring instituteand Guangdong Provincial Key Laboratory of Laboratory Animals, Guangzhou, 510633 China; 4 Institute of Animal Husbandry and Veterinary Medicine, Fujian Academy of Agricultural Sciences, Fuzhou, 350013 Fujian China; 5 Xingguo County Agricultural Technology Extension Center, Ganzhou, 341000 Jiangxi China; 6 XinyuYushui District Center for Agricultural Sciences, Xinyu, 338000 Jiangxi China

**Keywords:** Goose astrovirus genotypes 2, Real-time, Reverse transcription recombinase polymerase amplification, Point-of-care diagnosis

## Abstract

**Background:**

Goose astrovirus (GoAstV) is an important pathogen that causes joint and visceral gout in goslings. It has been circulating in many provinces of China since 2017. Goose astrovirus genotypes 2 (GoAstV-2) is the main epidemic strain, and its high morbidity and mortality have caused huge economic losses to the goose industry. An accurate point-of-care detection for GoAstV-2 is of great significance. In this study, we developed a real-time reverse transcription recombinase polymerase amplification (RT-RPA) method for the on-site detection of GoAstV-2 infection.

**Results:**

The real-time RT-RPA reaction was carried out at a constant temperature of 39 °C, and the entire detection time from nucleic acid preparation to the end of amplification was only 25 min using the portable device. The results of a specificity analysis showed that no cross-reaction was observed with other related pathogens. The detection limit of the assay was 100 RNA copies/μL. The low coefficient of variation value indicated excellent repeatability. We used 270 clinical samples to evaluate the performance of our established method, the positive concordance rates with RT-qPCR were 99.6%, and the linear regression analysis revealed a strong correlation.

**Conclusions:**

The established real-time RT-RPA assay showed high rapidity, specificity and sensitivity, which can be widely applied in the laboratory, field and especially in the resource-limited settings for GoAstV-2 point-of-care diagnosis.

## Introduction

Astroviruses are small, single-stranded, nonenveloped RNA viruses of the family *Astroviridae* that cause multiple illnesses, including gastroenteritis and diarrhea in humans, poor feed conversion and growth inhibition in turkeys, hepatitis in ducklings, and short stature syndrome and nephritis in chickens and pigeons [1–3]. According to the different species of origin, *Astroviridae* is classified into *Mamastrovirus* and *Avastrovirus*, which infect mammalian and avian species, respectively [4]. The *Avastrovirus* genus is divided into three species (*Avastrovirus 1*, *Avastrovirus 2*, and *Avastrovirus 3*), which are recognized by the International Committee on Taxonomy of Viruses [5, 6].

A novel disease with gout occurred in a geese flock of 1-week-old goslings in Anhui Province in 2015 [3]. By 2022, The disease had spread rapidly to other provinces in China, including Shandong, Fujian, Jiangsu, Zhejiang, Henan, Liaoning, Guangdong, Hunan, Sichuan, Heilongjiang, Inner Mongolia, Jiangxi, and Guangxi [7–9]. By viral isolation, propagation, high throughput sequencing, and phylogenetic tree analysis, the disease was indentied to be caused by a novel goose astrovirus (GoAstV) belonging to *Avastrovirus 3* since 2017 [8, 10]. GoAstV has two genotypes: GoAstV-1 and GoAstV-2, but the main epidemic strain causing gout, swelling and hemorrhage of kidneys, and urate in viscera in goslings was GoAstV-2 [11]. The infection of GoAstV-2 also resulted in 80% morbidity and 50% mortality in some goose farms, causing tremendous economic losses to the goose industry [12]. Therefore, a specific and simple assay for rapid detection of GoAstV-2 is important for epidemiological investigation and control of the disease.

Virus isolates are traditionally identified using methods that are inconvenient and have low sensitivity [13]. To rapidly detect the GoAstV, several molecular diagnostic methods had been reported, such as conventional RT-PCR [13], SYBR Green I real-time PCR [14], and one-step real-time RT-PCR based on TaqMan [15–17]. Due to their high sensitivity and specificity, these methods were widely applied in the laboratory by researchers; however, expensive or complicated equipment with temperature cycling is required to use these technologies, limiting their application on goose flocks. An immunochromatographic strip assay based on monoclonal antibodies was also developed for GoAstV detection because of its rapid and simple use [18], but the assay had lower sensitivity than methods based on nucleic acid amplification. In recent decades, isothermal amplification has been favored by most scientists, and a loop-mediated isothermal amplification (LAMP) method was established to diagnose the disease, but six primers were required in the RT-LAMP reaction for GoAstV-2 diagnosis, increasing the difficulty of primer design, and required high temperatures and long processing, as the RT-LAMP assay was performed at 60 °C for 60 min and terminated at 80 °C for 10 min [10, 19, 20].

Recombinase polymerase amplification (RPA) is considered an alternative nucleic acid detection technology to PCR that depends on three enzymes containing a recombinase polymerase, a single-stranded binding protein, and a DNA polymerase [21]. The RPA assay amplification can be performed in 10–20 min at a constant temperature of 37–42 °C. The RPA products can be verified by gel electrophoresis [22], visualized by lateral flow dipstick (LFD) [23], or detected using fluorescence signal analysis based on a probe [24]. Currently, the RPA technique has been successfully applied for the detection of various pathogens in humans, animals, plants, and foods [25–28].

To date, GoAstV-2 detection using real-time RT-RPA assay has not been reported. The aim of this study was to establish a rapid, specific, sensitive, and convenient real-time RT-RPA assay for GoAstV-2 detection in resource-limited farms, providing an alternative tool for disease diagnosis and prevention.

## Results

### Screening of the real-time RT-RPA primer–probe sets

**Fig. 1:**
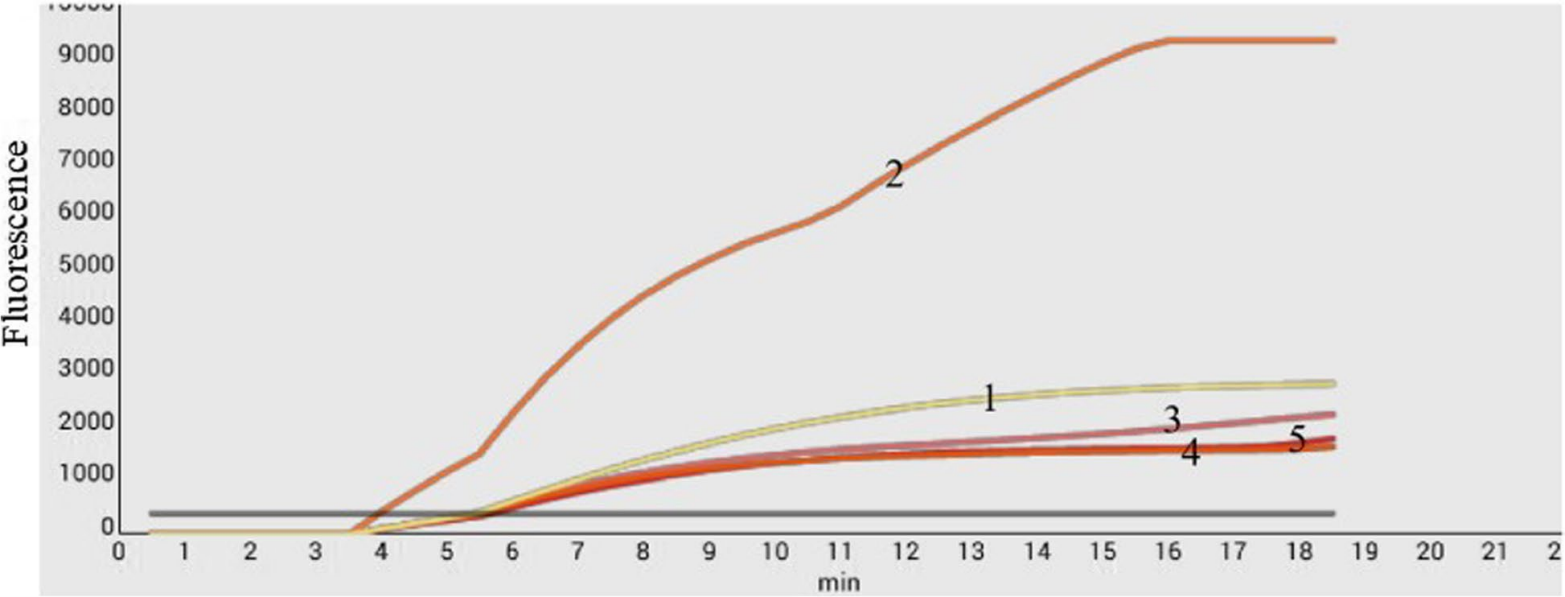
Screening of the primer–probe set. Two probes and five pairs of primers were designed and screened. Only probe 1 and primers F2/R2 generated strong fluorescent signals and therefore were used in the development of the real-time RT-RPA assay. 1: probe 1 and primer F1/R1; 2: probe 1 and primer F2/R2; 3: probe 1 and primer F3/R3; 4: probe 2 and primer F4/R4; 5: probe 2 and primer F5/R5

Two probes and five pairs of primers were designed based on the ORF2 sequence and evaluated using the GoAstV-2 RNA template and fluorescent RT-RPA kit. As shown in Fig. 1, the amplification curves were successfully observed using the primers F1/R1, F2/R2, or F3/R3 with probe 1, as well as primers F4/R4 or F5/R5 with probe 2. However, probe 2 and the suitable primer sets generated low fluorescent signals, resulting in low sensitivity and poor specificity. Therefore, probe 1 and primer F2/R2 were used for the development of the RT-RPA assay because they produced high fluorescent signals and a standard curve.

### Specificity, sensitivity, and reproducibility analysis of the GoAstV-2 real-time RT-RPA assay

**Fig. 2:**
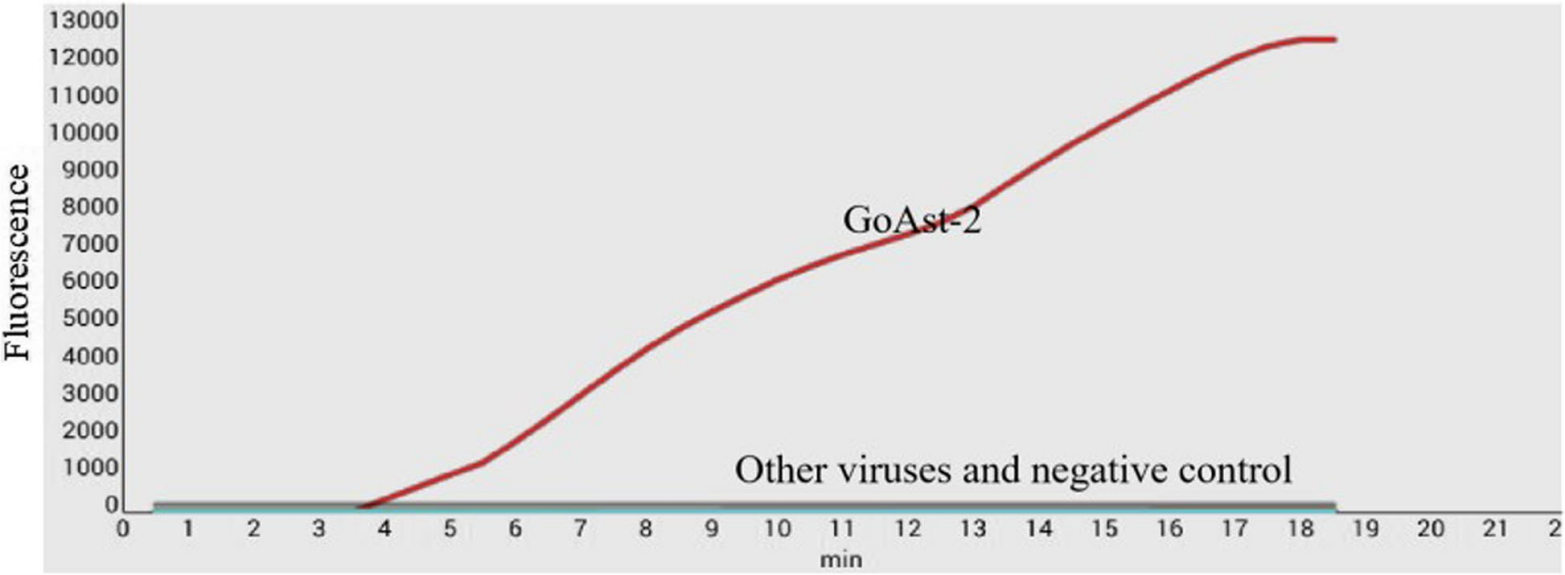
Specificity analysis of the real-time RT-RPA assay. The nucleic acids of other viruses, including goose astrovirus 2 (GoAstV-2), goose astrovirus 1 (GoAstV-1), goose parvovirus (GPV), duck tembusu virus (DTMUV), and duck reovirus (DREV), were used as templates to assess the specificity of the real-time RT-RPA assay. Only GoAstV-2 generated an amplification curve, and no non-specific fluorescent signals were observed with other viruses or negative control

The specificity analysis of the GoAstV-2 real-time RT-RPA assay was performed using the nucleic acids of GoAstV-2, GoAstV-1, DTMUV, GRV, GPV, AIV, NDV, and FAdV as templates. The results showed that only GoAstV-2 generated a strong, specific fluorescent signal. No amplification curve was observed for the other avian viruses used in the study or the negative control (Fig. 2). In addition, the same assay was carried out three times, and the results were similar, indicating good specificity of the developed real-time RT-RPA for GoAstV-2 detection.

**Fig. 3:**
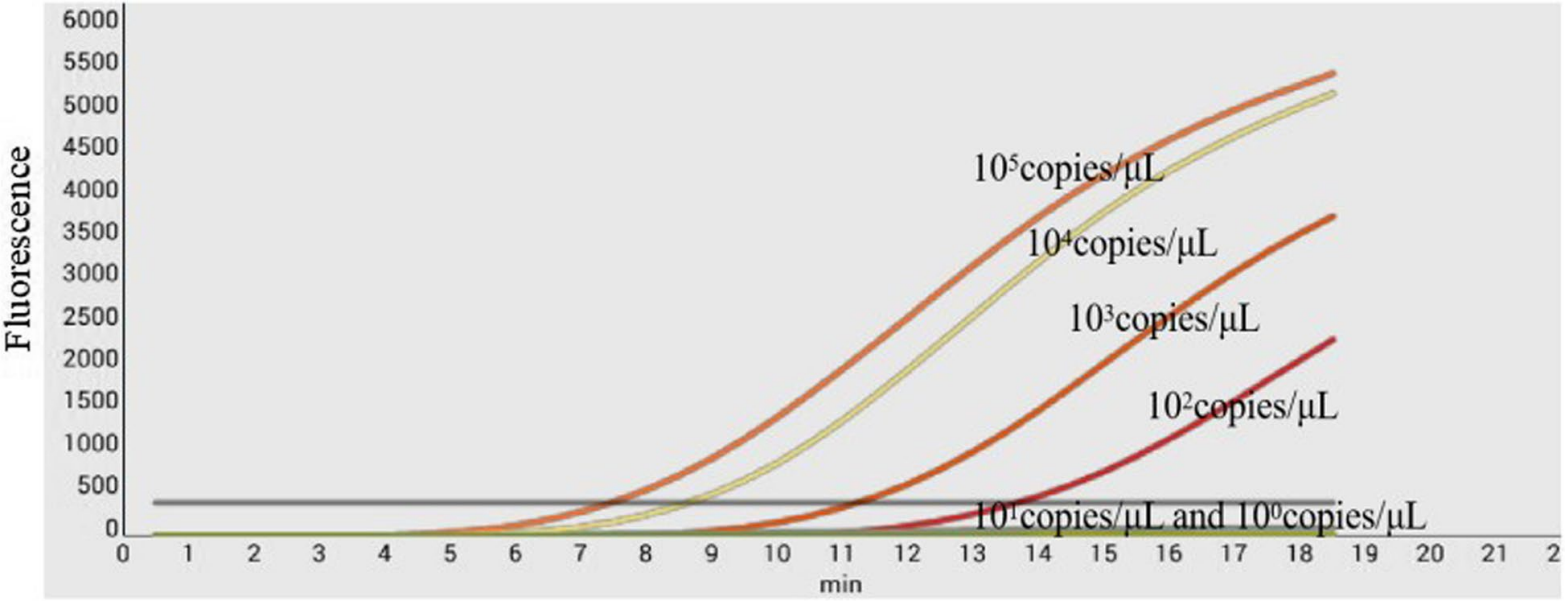
Sensitivity analysis of the real-time RT-RPA assay. Serial ten-fold dilutions of the RNA standard ranging from 10^5^ to 10^0^ copies/μL were analyzed using the real-time RT-RPA assay. The results showed that the RNA standard from 10^5^ to 10^2^ copies/μL was determined to be positive between 4 and 14 min and no fluorescent signals were detected from 10^1^ and 10^0^ copies/μL of RNA standard, demonstrating that the detection limit was 10^2^ copies/μL

The different diluted copy numbers of the constructed RNA standards were used as templates to assess the detection limit of the real-time RT-RPA assay. As shown in Fig. 3, a fluorescence signal was observed and a typical amplification curve appeared for the RNA standard from 10^5^ copies/μL to 10^2^ copies/μL, whereas no signal was obtained from 10^1^copies/μL to 10^0^ copies/μL of the RNA standard in the study, showing that the limit of detection of the assay was 10^2^ copies/μL.

To determine the intra-assay reproducibility, six different concentrations ranging from 10^5^ copies/μL to 10^0^ copies/μL were analyzed with five replicates in each reaction. The same template was analyzed to evaluate the inter-assay repeatability on five different days. For the 10^5^–10^2^ copies/μL RNA standards, the positive reaction rate was 100% in 10/10 runs, and 0/10 runs generated a fluorescence signal at concentrations of 10 copies/μL and 1 copies/μL. The CV values of the intra- and inter-assay were < 5.5% (Table 1), showing high reproducibility and stability of the real-time RT-RPA assay for GoAstV-2 detection.

**Table 1:** Reproducibility analysis of the real-time RT-RPA assay using different RNA standards

C (copies/μL)	Intra-assay	CV (%)	Inter-assay	CV (%)
	TT value mean ± SD		TT value mean ± SD	
1 × 10^5^	7.00 ± 0	0	6.92 ± 0.37	5.35
1 × 10^4^	8.24 ± 0.13	1.63	8.44 ± 0.31	3.71
1 × 10^3^	10.72 ± 0.38	3.58	10.92 ± 0.37	3.39
1 × 10^2^	13.58 ± 0.38	2.82	13.72 ± 0.38	2.79

*TT* threshold time

### Application on clinical samples

**Fig. 4:**
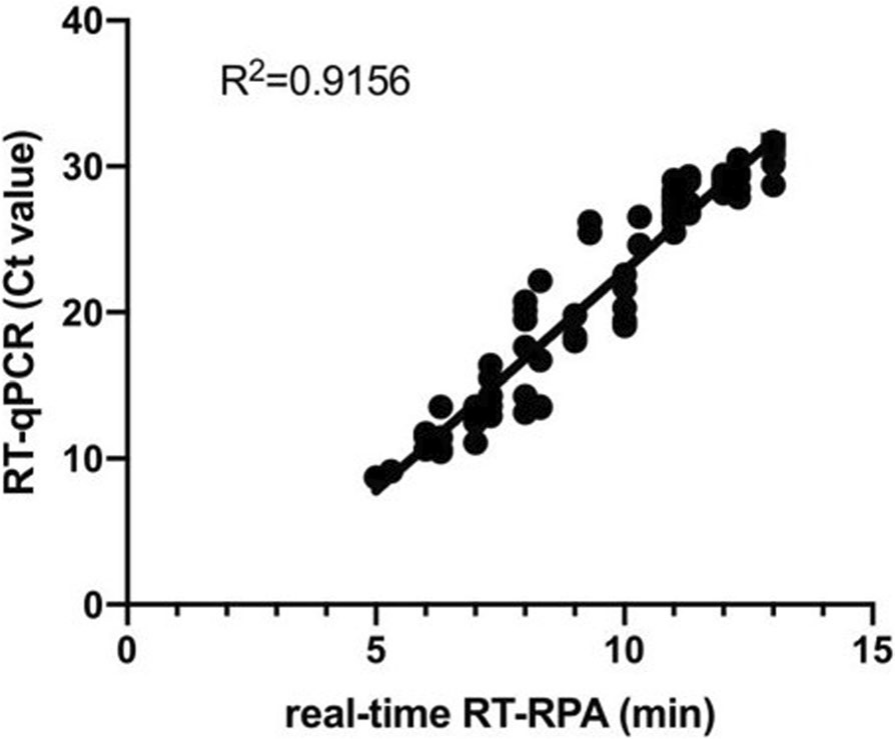
Clinical performance using the real-time RT-RPA assay and RT-qPCR assay. For the 123 positive samples, the linear regression between the threshold time (TT) of real-time RT-RPA (x-axis) and Ct value of RT-qPCR (y-axis) was analyzed using GraphPad Prism 8.0 software. The R2 value was 0.9156, showing a good correlation

Sixty tissue samples artificially infected with GoAstV-2, 120 clinical tissue samples, and 90 cloacal swabs from geese farms collected in 2021–2022 were used to further validate the performance of the established GoAstV-2 RT-RPA assay. The nucleic acid extraction of the samples with lysis buffer and commercial extraction kits were analyzed by the GoAstV-2 real-time RT-RPA assay and RT-qPCR method, respectively. All of 60 tissue samples artificially infected with GoAstV-2 were positive using the two methods. Thirty-eight of 120 clinical tissue samples and 25 of 90 cloacal swabs were detected to be positive by the real-time RT-RPA assay. Of the 210 samples analyzed by the RT-qPCR assay, all the positive samples by RT-RPA were also tested to be positive. Only one negative tissue sample by the RT-RPA assay was determined to be weakly positive by RT-qPCR, with a Ct value of 33.345 (Table 2). The nucleic acid of this tissue sample, which was extracted using commercial kits, was then analyzed by real-time RT-RPA, and an amplification curve was observed, suggesting that the crude nucleic acid extraction could contain impurities affecting RPA amplification for individual weakly positive samples. 124 of 270 samples were confirmed as positive by sequencing. Among the positive samples, the linear regression was analyzed by GraphPad Prism 8.0, and the result demonstrated that both the threshold time of RT-RPA and the Ct values of RT-qPCR correlated well (Fig. 4).

**Table 2:** Coincidence rate of clinical samples using the real-time RT-RPA assay and RT-qPCR assay

	Detection rate of positive samples		Coincidence rate
	Real-time RT-RPA	RT-qPCR	
Artificially infected samples	60/60 (100%)	60/60 (100%)	99.6%
Clinical tissue samples	38/120 (31.67%)	39/120 (32.5%)	
Cloacal swabs	25/90 (27.78%)	25/90 (27.78%)	

Coincidence rate = (123 + 146)/270*100%

## Discussion

In recent years, the disease caused by GoAstV was discovered in goslings and rapidly became prevalent in most areas of China. Its high morbidity and mortality had become a serious threat to the goose industry [29]. Accordingly, it is very beneficial to establish a rapid, low-resource, and efficient method for GoAstV-2 point-of-care diagnosis. To our knowledge, this is the first report for the rapid detection of GoAstV-2 using a real-time RT-RPA assay targeting the ORF2 gene. The developed RT-RPA reaction was completed at an isothermal temperature of around 39 °C for less than 20 min without the requirement of a complicated procedure and expensive instruments.

The design and screening of the primers and probe are key to determining the amplification efficiency and to avoiding false positive and false negative results [30]. The optimal probe–primer combination from two probes and 10 primers was selected to amplify the ORF2 gene, which were used as the target sequence because they were reported to be highly conserved in several previously developed methods based on nucleic acid amplification, but a few sequence variations still appeared in the ORF2 genes of GoAstV-2 strains. The RPA assay was reported to be able to tolerate 5–9 target mismatches with little influence on the results of the assay [31, 32]. The probe–primer set used in this study had a strong fluorescence signal and no cross-reaction with other related pathogens, demonstrating good amplification efficiency and high specificity of the assay.

The limit of detection of the GoAstV-2 real-time RT-RPA assay was 100 copies/μL using an in vitro transcribed RNA standard, which was ten-fold higher than the 10 copies/L required for the qLAMP assay and a bit higher than the 33.4 copies/μL required for TaqMan-based RT-PCR [10, 17]. The developed real-time RPA assay is more suitable for point-of-care diagnosis in the field in terms of a shorter reaction time, lower amplification temperatures, and simple equipment. Other studies showed that a higher sensitivity was observed in RPA assays compared with LAMP, universal PCR, and qPCR assays [26, 33, 34]. The sensitivity in the study can be improved by further optimizing the conditions in the future.

Some researchers had constructed a mobile laboratory for DNA and RNA virus point-of-care diagnostics in the field using the RPA method [35, 36]. The nucleic acid extraction was a challenge to detect in the field with a shortage of equipment resources, as most commercial extraction kits were more suitable for the laboratory. The RPA assay was shown to be more tolerant to inhibitors than other nucleic acid amplification methods [37]. In this study, the lysis buffer containing all the required components was used to quickly extract the nucleic acid for clinical sample detection. A total of 270 clinical samples were simultaneously tested by RT-qPCR and RT-RPA assays, and only one weak positive sample was detected to be a false negative by the real-time RT-RPA. The results showed a 99.6% concordance, suggesting that individual samples may be affected by the inhibitor in the quick crude nucleic acid extraction in the RT-RPA reaction. Linear regression analysis showed that the R2 value was 0.9156, indicating that the developed RT-RPA method could be applied for semi-quantitative detection of GoAstV-2.

Serological identification and molecular detection methods have been developed to detect GoAstV-2 [16, 20, 38, 39]. These diagnostic techniques have the advantage of high accuracy, specificity, and repeatability, but these methods are usually labor-intensive and time-consuming and require skilled professionals and special instruments, limiting their widespread use in remote areas. The RT-RPA technique not only was highly specific and sensitive but also was tolerant to primer mismatch, was tolerant to reaction inhibitor, used portable equipment, and had a shorter detection time, which was only 30 min from nucleic acid preparation to RT-RPA reaction. The main reagents were provided in a freeze-dried pellet that is easy to preserve, providing a point-of-care detection for GoAstV-2 in the field.

## Conclusion

A real-time RT-RPA assay with fast nucleic acid extraction was developed for GoAstV-2 on-site detection. The assay proved to be rapid, accurate, specific, and convenient, and can be an alternative diagnostic tool for surveillance and control of the disease, especially in low-resource settings or in the field.

### Materials and methods

#### Virus strains

The novel goose astrovirus genotypes 2 (GoAstV-2) was isolated from goose kidneys with gout. Goose kidney tissue samples were homogenized with sterile phosphate-buffered saline (PBS, PH7.2) to a 10% suspension (w/v) and centrifuged at 4℃ at 5000 g for 15 min. the supernatant that filtered through a 0.22 μm filter was inoculated in 11-day-old healthy goose embryos through the chorioallantoic membrane at 37℃ for 6 days. The collected chorioallantoic membrane homogenates were detected using real-time RT-PCR assay and the positive samples were sequenced for comfirmation [40]. GoAstV-2 JX01/China/2021 strain (GenBank: MZ576222.1), Goose astrovirus genotypes 1 (GoAstV-1) JXGZ strain (GenBank: OL762471.1), goose parvovirus (GPV) SYG41-50 strain, Duck tembusu virus (DTMUV) FJMH220 strain, and duck reovirus (DREOV) CA strain were preserved in our laboratory and stored at − 80 °C before use. The nucleic acids of Newcastle disease virus (NDV) F48E9 strain, avian influenza virus (AIV) H7N2 subtype, and fowl adenovirus serotype 4 (FAdV-4) were obtained from Guangdong Laboratory Animals Monitoring Institute.

#### Design of real-time RT-RPA probes and primers

The ORF2 complete gene sequences of GoAstV-2 were downloaded from GenBank and aligned using the MegAlign software. According to the design manual from TwistDx Co. Ltd., two exo probes were designed and carefully screened based on the conserved region of the ORF2 sequence. The TwistAmpexo probe length was 46–52 nucleotides. A pair of T residues in close proximity to one another (with only 1–5 intervening nucleotides) were found in a probe sequence, and the relevant T residues were replaced by dT-fluorophore residues and dT-quencher residues. One base was replaced by the THF residue, and the 3’-end was blocked with a C3-spacer [41, 42]. Three pairs of primers were designed to match probe 1 and two pairs of primers were suitable for probe 2. The sequences of all primers and probes are listed in Table 3 and were synthesized by Sangon Biotech (Shanghai, China) Co. Ltd.

**Table 3:** The sequences of primers and probes used in this study

Method	Name	Sequence (5′–3′)	Amplicon size (bp)	Source
Real-time RT-RPA	GoAstV-F1	TAATGACAAAATGACTACCACAATAACACT	161	this study
	GoAstV-R1	TATATTGTGAAGCCCTTATACTTAGAGGAG		
	GoAstV-F2	CGTTAATGACAAAATGACTACCACAATAAC	165	
	GoAstV-R2	GTTATATTGTGAAGCCCTTATACTTAGAGG		
	GoAstV-F3	AAAGAAGTGAAAGGTTTGAAGAAAAGAGTA	241	
	GoAstV-R3	CAAGGCGGATATGCAGCTTTCTGATCCTCC		
	GoAstV-probe1	CAACAGACACACTCGACCGGAAGCATAAA[FAM-dt] [THF]C[BHQ1-dT] TCACAAATCCACTC (C3-spacer)		
	GoAstV-F4	AGGTCAAGATACAATGCAAATATAACCTTC	126	
	GoAstV-R4	AAAAATAACTCCTGTTTGTGAAAGTGTCAT		
	GoAstV-F5	GAATACCACAGGCAGACTCCAGGTCAAGAT	141	
	GoAstV-R5	TAACTCCTGTTTGTGAAAGTGTCATAACAG		
	GoAstV-probe2	ATGTTGGCTATCGTGGAAGGACTTCAACA[FAM-dt] [THF]A[BHQ1-dT] TTACACTTGGGACA (C3-spacer)		
RT-PCR	GoAstV-F	CAGTATCTGGCATCGCCTCAT	406	
	GoAstV-R	CCTGGGAACAGAACCTGAACT		
RT-qPCR	qF	GGCCAATATTCAACAACA	172	Chunhe Wan et al., 2019
	qR	CCTTCCTTATTGACACAAG		
	qP	TGTGTAATGTCTGGCTCACCCA		

#### Construction of the RNA standard

The partial sequences (406 bp) of the nucleocapsid gene was amplified by the primers GoAstV-F and GoAstV-R using a one-step RT-PCR kit (Takara, Dalian, China). The amplification product was purified by a gel extraction kit (Omega, USA) and inserted into the pGEM-T vector (Promega, USA). The positive recombinant plasmid pGEM-T-cap was sequenced by Sangon Biotech (Shanghai, China) and linearized by SalI (Takara, Dalian, China). The purified linear DNA was in vitro transcribed to RNA using a T7 RiboMAX™ Express RNAi System (Promega, USA) following the manufacturer’s manual. The double-stranded RNA was purified using a Qiagen Viral RNA mini kit (Qiagen, Germany). The RNA standard concentration was determined using a NanoDrop 2000 spectrophotometer (Thermo Fisher, USA), and the copy number was calculated using a previously described formula [21].

#### GoAstV-2 real-time RT-RPA assay

The real-time RT-RPA reaction was performed in a 50 μL volume with a fluorescent RT-RPA kit (Zongce, Hangzhou, China). The reaction tube contained lyophilized enzyme pellets comprising 25 μL of rehydration buffer (A buffer), 2 μL of each primer (10 μmol/L), 0.6 μL of the RT-RPA probe (10 μmol/L), 2.5 μL of magnesium acetate (B buffer), 2 μL of the RNA/DNA template, and 15.9 μL of RNase-free H_2_O and incubated at 39 °C for 20 min using a Deaou-308C instrument (DEAOU Biotechnology, Guangzhou, China). The fluorescence signal was collected and the time of the amplification curve was calculated based on the threshold. If the amplification curve was generated within 20 min, the sample was set as positive; otherwise, the sample was negative.

#### Specificity analysis of the real-time RT-RPA assay

The nucleic acid of GoAstV-2 and other avian viruses, including GoAstV-1, DTMUV, DREOV, GPV, AIV, NDV, and FAdV, and distilled water were tested to evaluate the specificity of the GoAstV-2 RT-RPA assay using the above mentioned procedure. GoAstV-2 RNA and distilled water were used as positive and negative controls, respectively.

#### Sensitivity and repeatability analysis of the real-time RT-RPA assay

To analyze the limit of detection of the GoAstV-2 RT-RPA assay, the in vitro transcribed RNA standard was serially diluted ten-fold in EASY Dilution solution ranging from 10^5^ copies/μL to 10^0^ copies/μL. RNase-free H_2_O, instead of the RNA standard, was tested as a negative control. All reactions were performed in triplicate.

To validate the repeatability of the method, six tenfold gradient dilutions (10^5^, 10^4^, 10^3^, 10^2^, 10^1^, and 10^0^ copies/μL) of RNA standard were carried out five times using the developed RT-RPA assay under optimized reaction conditions, and the coefficient of variation (CV) values of the intra- and inter-assay were calculated.

#### Clinical sample analysis using the RT-RPA and real-time RT-PCR assays

A total of 270 goose clinical samples, including 60 tissue samples artificially infected with GoAstV-2, 120 clinical tissue samples (kidney, spleen, and liver), and 90 cloacal swabs were collected from geese farms in Jiangxi Province, China. All goslings used in the experiment were euthanized by intravenous injection of sodium pentobarbital (100 mg/kg body weight). The tissue samples were homogenized with phosphate-buffered saline (PBS; pH 7.2) at a ratio of tissue: PBS of 1:10 and centrifuged at 4 °C at 5000 g for 15 min. The supernatants were collected and stored at − 80 °C. The cloacal swabs were placed in a 2 mL tube with 1 mL of PBS and mixed. All the samples were treated with lysis buffer at a ratio of 1:1 and centrifuged, and then were used as templates in the real-time RT-RPA assay. To further validate the results of the assay detection, all the samples were also tested by real-time RT-PCR as previously described [17]. Total RNA and DNA were also extracted from the supernatants of the tissue homogenates and the cloacal swabs using a TIANamp Virus Genomic DNA/RNA kit (Tiangen Biotech, Beijing, China) in accordance with the manufacturer’s protocol and stored at − 80 °C until used for RT-qPCR.

## Data Availability

All data generated or analyzed during this study are included in this published article.

## Abbreviations

RT-RPA: Reverse transcription recombinase polymerase amplification
GoAstV-2: Goose astrovirus 2
GoAstV-1: Goose astrovirus 1
GPV: Goose parvovirus
DTMUV: Duck tembusu virus
DREOV: Duck reovirus
NDV: Newcastle disease virus
AIV: Avian influenza virus
FAdV: Fowl adenovirus
RT-qPCR: Real-time reverse transcription polymerase chain reaction
qLAMP: Real-time loop-mediated isothermal amplification
