# Correction: Rapid detection of goose astrovirus genotypes 2 using real-time reverse transcription recombinase polymerase amplification

**DOI:** 10.1186/s12917-023-03818-7

**Published:** 2023-11-23

**Authors:** Haiqin Li, Yujun Zhu, Chunhe Wan, Zhangzhang Wang, Lei Liu, Meifang Tan, Fanfan Zhang, Yanbing Zeng, Jiangnan Huang, Chengcheng Wu, Yu Huang, Zhaofeng Kang, Xiaoquan Guo

**Affiliations:** 1 Institute of Animal Husbandry and Veterinary Medicine, Jiangxi Academy of Agricultural Sciences, Nanchang, Jiangxi 330200 China; 2 Jiangxi Provincial Key Laboratory for Animal Health, College of Animal Science and Technology, Jiangxi AgriculturalUniversity, Nanchang, China; 3 Guangdong laboratory animals monitoring instituteand Guangdong Provincial Key Laboratory of Laboratory Animals, Guangzhou, 510633 China; 4 Institute of Animal Husbandry and Veterinary Medicine, Fujian Academy of Agricultural Sciences, Fuzhou, 350013 Fujian China; 5 Xingguo County Agricultural Technology Extension Center, Ganzhou, Jiangxi 341000 China; 6 XinyuYushui District Center for Agricultural Sciences, Xinyu, Jiangxi 338000 China

**Correction: BMC Vet Res 19, 232 (2023)**

10.1186/s12917-023-03790-2

Following publication of the original article [1], the authors would like to correct the corresponding author’s name from “Xiaoqiao Guo” to “Xiaoquan Guo”.
